# Supplementary material for: Selection signatures in goats reveal copy number variants underlying breed-defining coat color phenotypes
Source: PLoS Genet. 2019 Dec 16;15(12):e1008536. doi: 10.1371/journal.pgen.1008536 (PMC6936872; doi:10.1371/journal.pgen.1008536)
Supplement: S1 Fig — (PDF) [file pgen.1008536.s001.pdf]

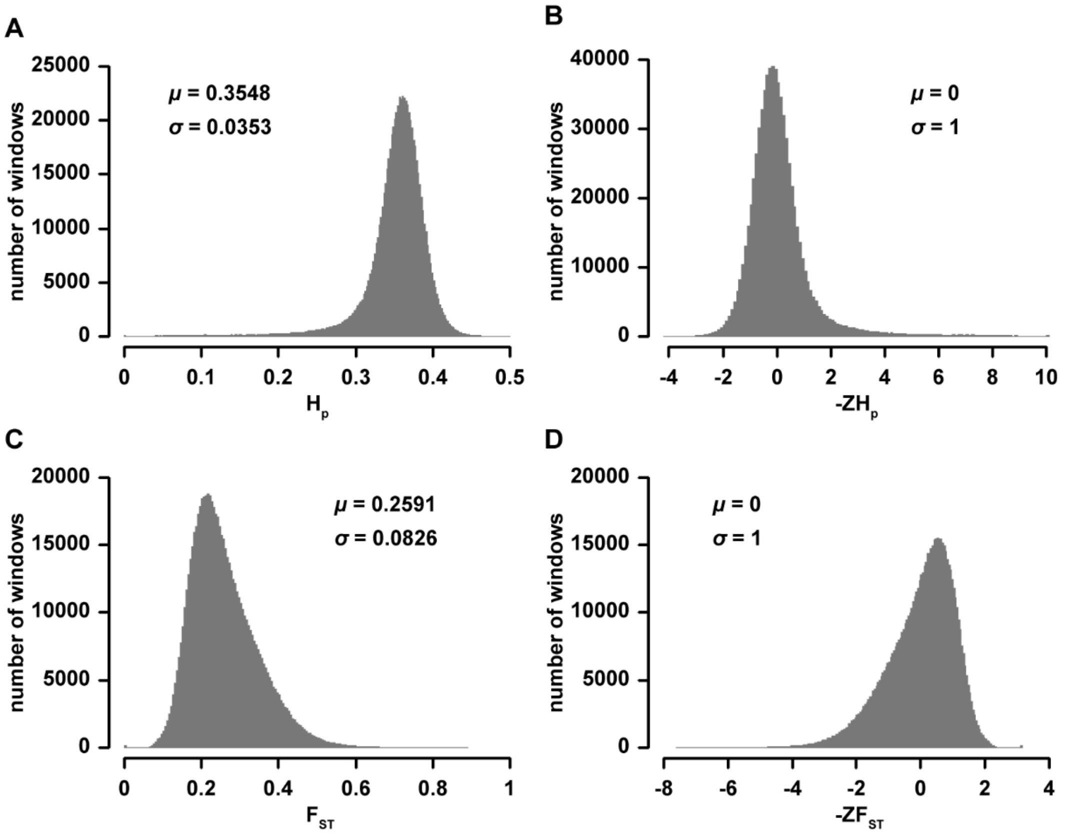

S1 Figure. Distributions of H_p, $-ZH_p$, F_{ST} and $-ZF_{ST}$. **A** The plot shows the distribution of all calculated Hp scores within a 150 kb window. **B** Standardized normal distribution of negative Z-transformed Hp scores. **C** Distribution of all calculated pairwise F_{ST} scores to bezoars within a 150 kb window. **D** Standardized normal distribution of negative Z-transformed F_{ST} values. Mean (μ) and standard deviation (σ) are indicated.
