# Supplementary material for: Selection signatures in goats reveal copy number variants underlying breed-defining coat color phenotypes
Source: PLoS Genet. 2019 Dec 16;15(12):e1008536. doi: 10.1371/journal.pgen.1008536 (PMC6936872; doi:10.1371/journal.pgen.1008536)
Supplement: S2 Fig — (PDF) [file pgen.1008536.s002.pdf]

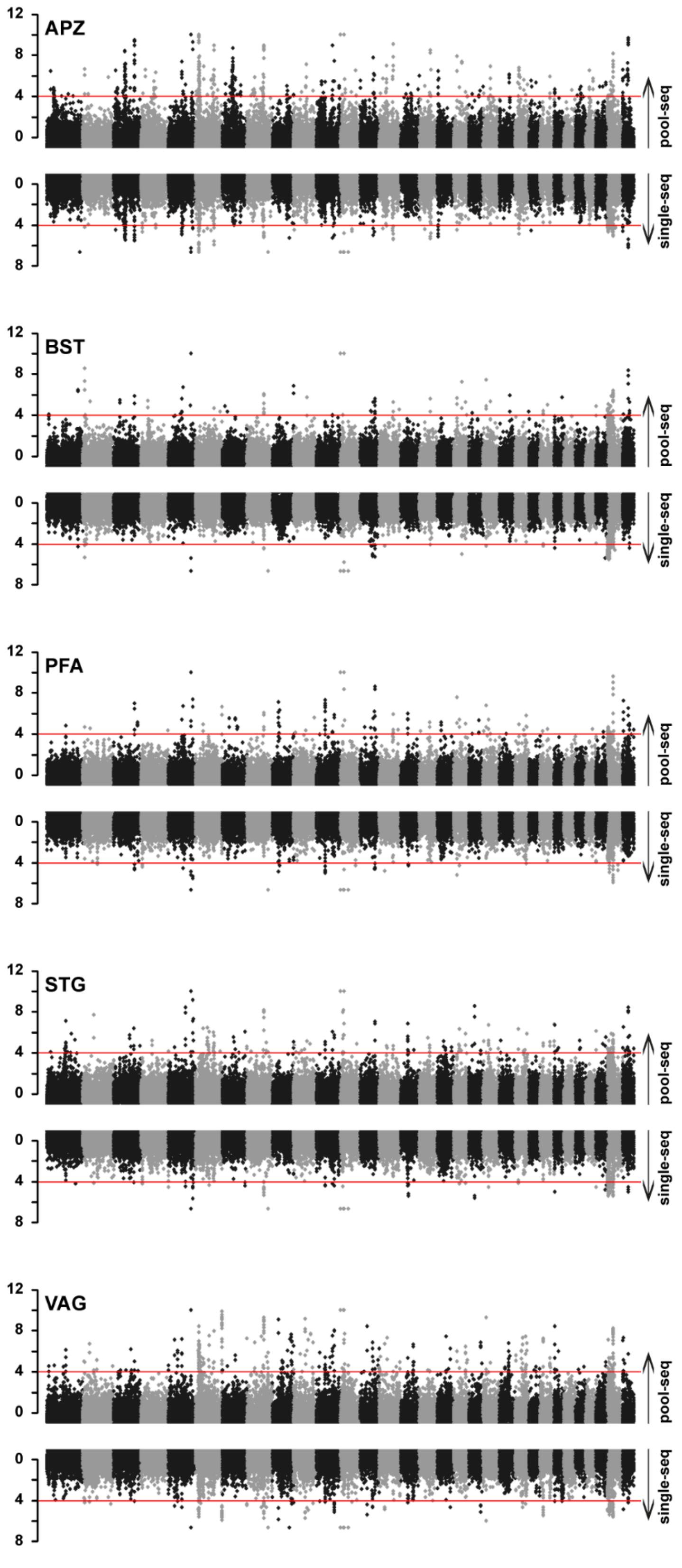


**S2 Figure.** Comparison of Manhattan plots with $-ZH_p$ scores between pool-seq data and 24 individually sequenced goat samples ("single-seq"). The red line indicates the chosen significance threshold of $-ZH_p = 4$. Each dot represents a 150 kb window. Each plot shows 29 autosomes and two unplaced scaffolds representing the X chromosome.
