# Supplementary material for: Selection signatures in goats reveal copy number variants underlying breed-defining coat color phenotypes
Source: PLoS Genet. 2019 Dec 16;15(12):e1008536. doi: 10.1371/journal.pgen.1008536 (PMC6936872; doi:10.1371/journal.pgen.1008536)
Supplement: S3 Fig — (PDF) [file pgen.1008536.s003.pdf]

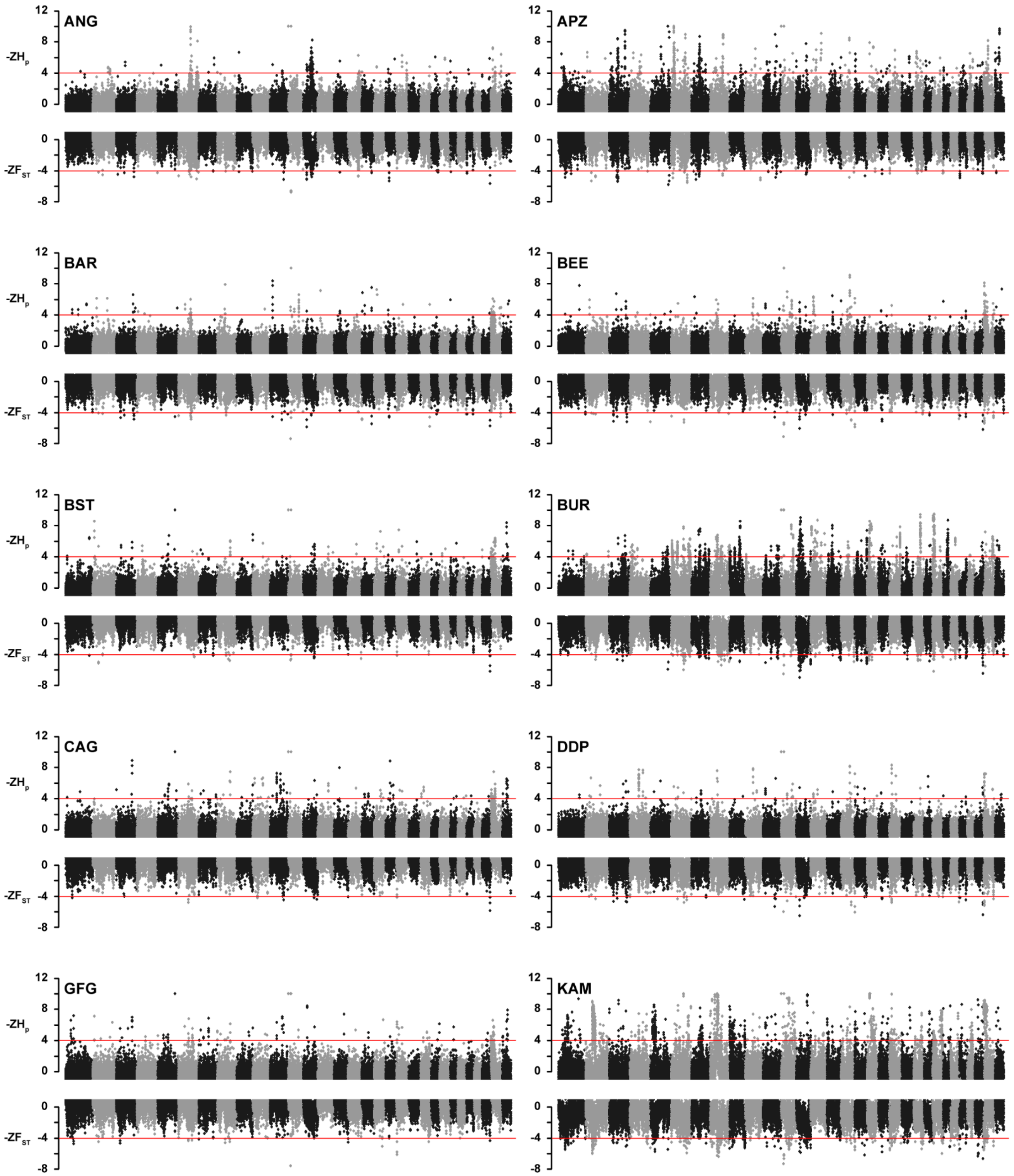
ANG
APZ
BAR
BEE
BST
BUR
CAG
DDP
GFG
KAM
$-ZH_p$
$-ZF_{ST}$
12
8
4
0
-4
-8

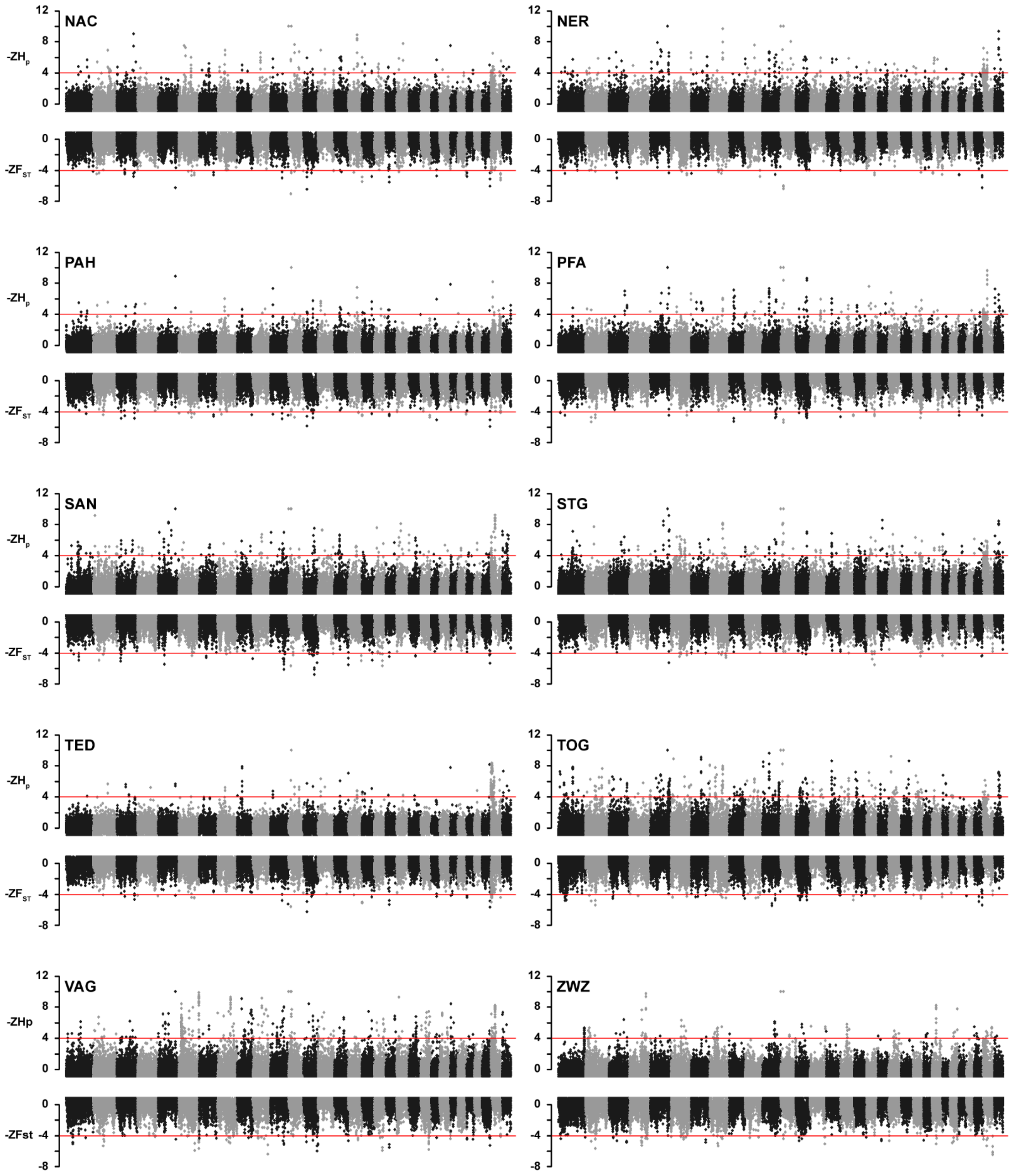


**S3 Figure.** Manhattan plots with $-ZH_p$ and $-ZF_{ST}$ scores of 20 diverse goat breeds. The red line indicates the chosen significance threshold of $-ZH_p = 4$ and $-ZF_{ST} = -4$. Each dot represents a 150 kb window. Each plot shows 29 autosomes and two unplaced scaffolds representing the X chromosome.
