# Supplementary material for: Selection signatures in goats reveal copy number variants underlying breed-defining coat color phenotypes
Source: PLoS Genet. 2019 Dec 16;15(12):e1008536. doi: 10.1371/journal.pgen.1008536 (PMC6936872; doi:10.1371/journal.pgen.1008536)
Supplement: S4 Fig — (PDF) [file pgen.1008536.s004.pdf]

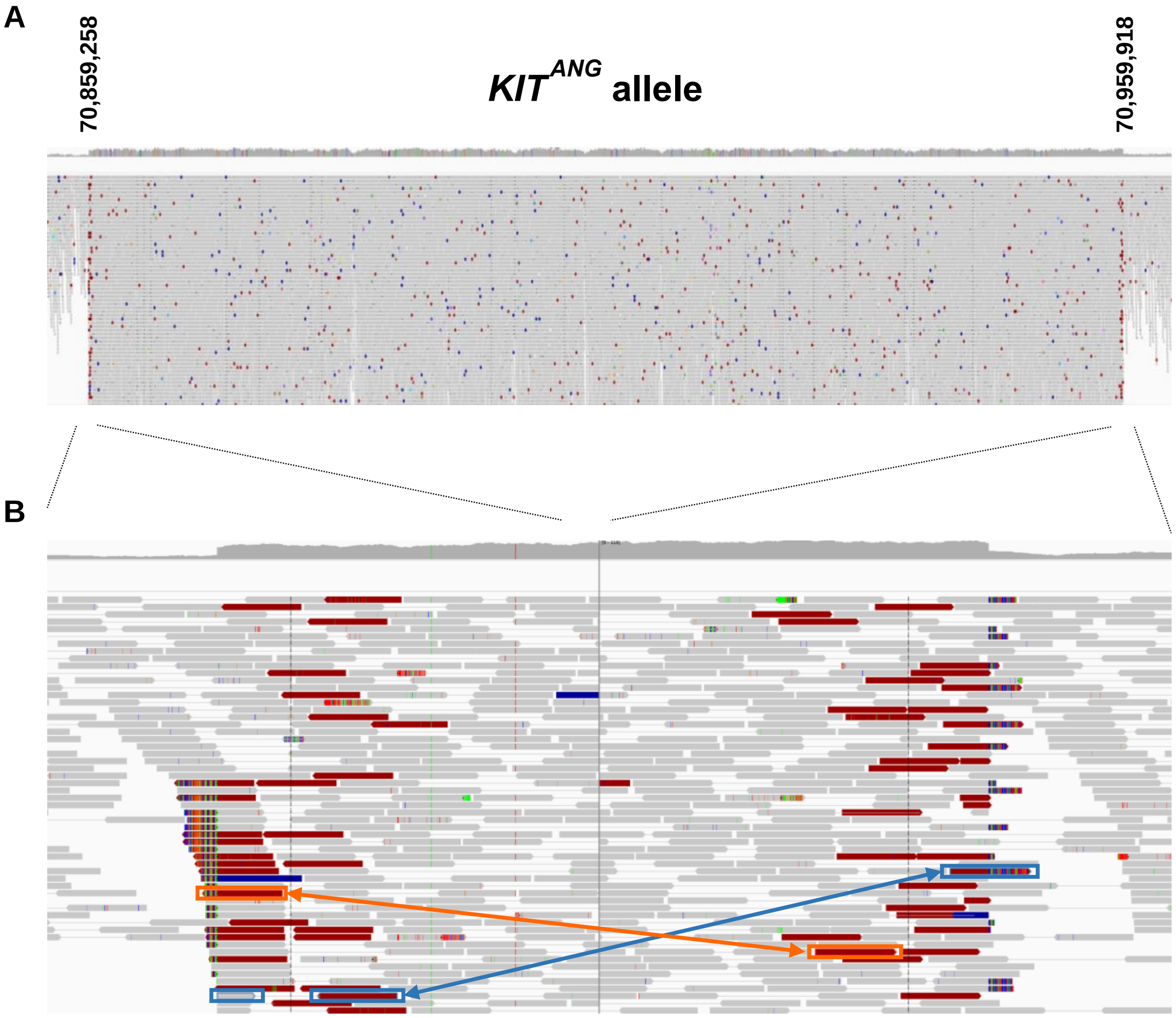

S4 Figure. Details of the CNV alleles. **A** IGV screenshot of the *KIT*^{ANG} allele derived from the whole genome sequence data of the pool of Pak Angora goats. The *KIT*^{ANG} allele contains 3 tandem copies of a ~100 kb sequence region. **B** Enlarged details of the breakpoints. Two read-pairs bridging individual copies of the amplified region are highlighted in blue and orange, respectively. The soft-clipped based at the left boundary align to the reference sequence at the right boundary of the amplified region. This confirms that the amplified copies are arranged in tandem in a head-to-tail orientation.

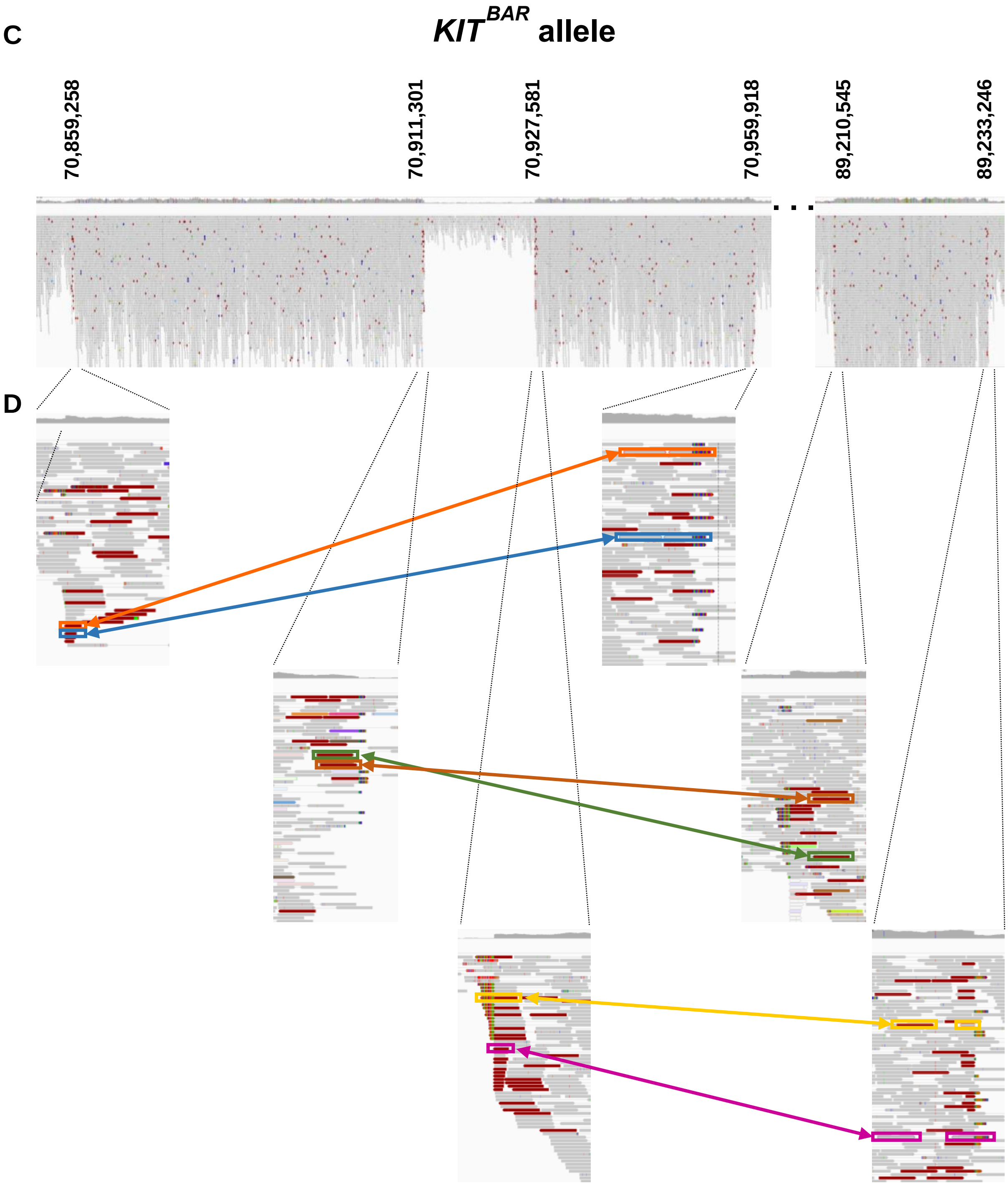

S4 Figure. Details of the CNV alleles. **C** IGV screenshot of the *KIT*^{BAR} allele derived from the whole genome sequence data of the pool of Barbari goats. The *KIT*^{BAR} allele contains 2 tandem copies of a ~100 kb sequence region. The breakpoints at 70,859,258 and 70,959,918 are identical to the *KIT*^{ANG} allele. A ~16 kb sequence element is deleted and replaced by two copies of a ~23 kb element derived from a chromosome fragment 19 Mb further distal. A few read alignments are visible in the “deleted” region spanning from 70,911,301 – 70,927,581. We speculate that these reads are due to the presence of one or two wildtype chromosomes in the pool-seq data of the Barbari goats. **D** Enlarged details of the breakpoints. Individual reads of read-pairs are highlighted in color. These read-pairs confirms that the amplified copies are arranged in tandem in a head-to-tail orientation.

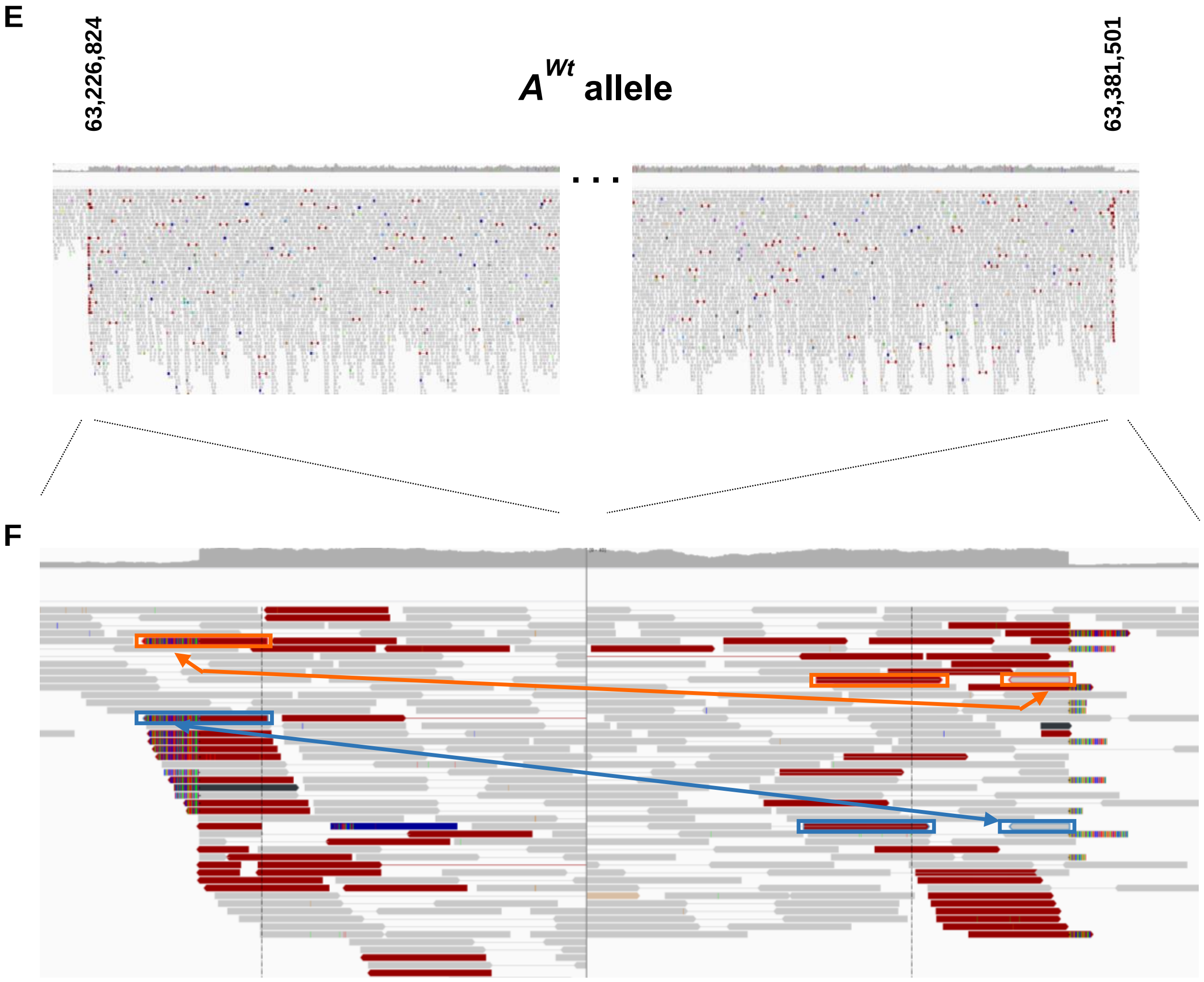

S4 Figure. Details of the CNV alleles. **E** IGV screenshot of the A^{Wt} allele derived from the whole genome sequence data on an individual Appenzell goat. The A^{Wt} allele contains 3 tandem copies of a ~155 kb sequence region. **F** Enlarged details of the breakpoints. Two read-pairs bridging individual copies of the amplified region are highlighted in blue and orange, respectively. The soft-clipped based at the left boundary align to the reference sequence at the right boundary of the amplified region. This confirms that the amplified copies are arranged in tandem in a head-to-tail orientation.

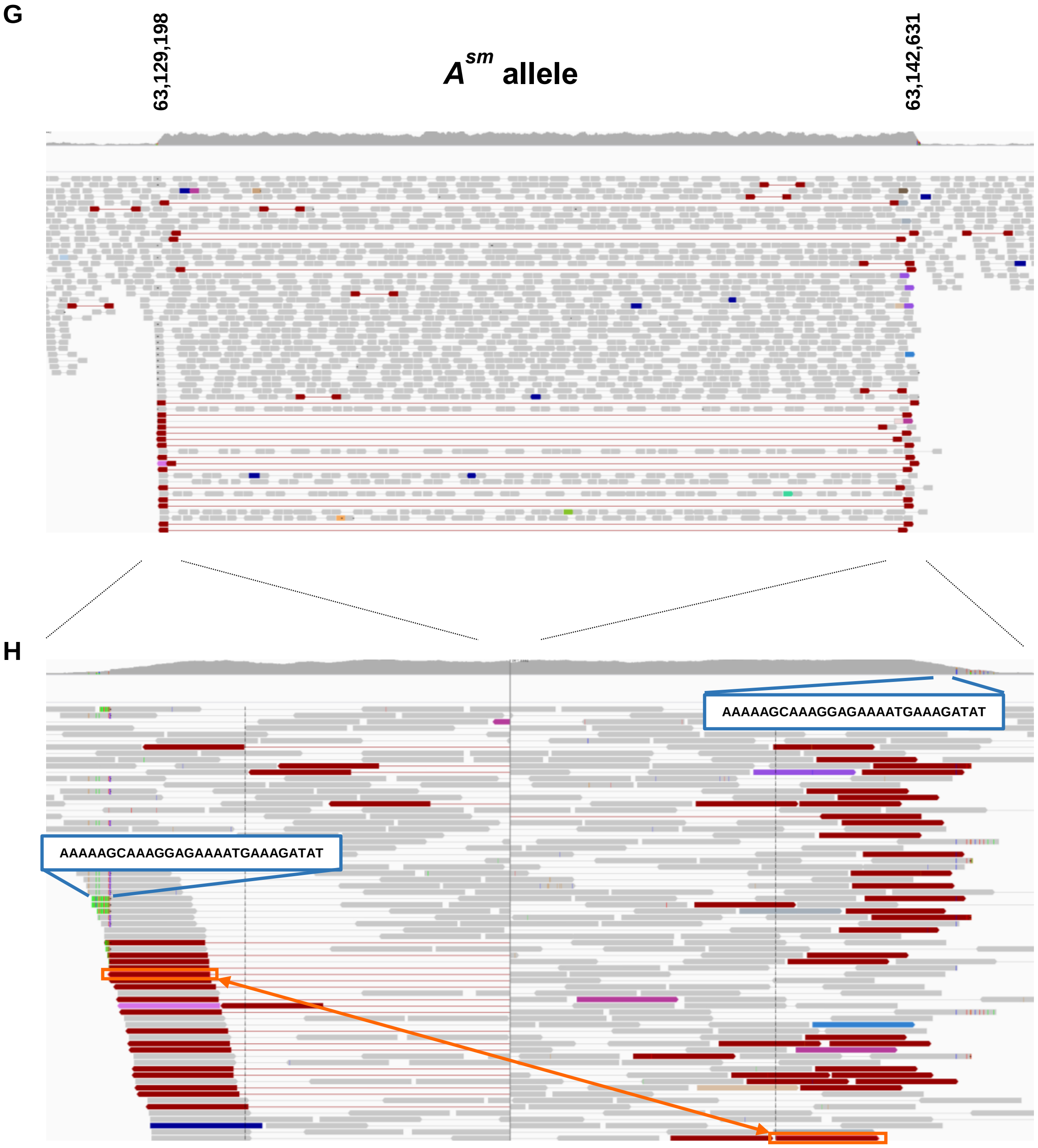

S4 Figure. Details of the CNV alleles. **G** IGV screenshot of the A^{sm} allele derived from the whole genome sequence data on an individual Grisons Striped goat. The A^{sm} allele contains 8 tandem copies of a ~13 kb sequence region. The illumina sequence reads are displayed as read-pairs. Several read-pairs that span from one into the next copy of the CNV are highlighted in red color. Only a subset of the reads in the amplified region is shown. **H** Enlarged details of the breakpoints. A read-pair spanning the breakpoint of the amplified region is highlighted in orange. Note that the two reads are facing outwards. The breakpoints of the A^{sm} CNV are located in a low-complexity/repetitive region. The 28 soft-clipped bases of one read at the left boundary perfectly align to the reference sequence at the right boundary of the amplified region. This confirms that the amplified copies are arranged in tandem in a head-to-tail orientation.

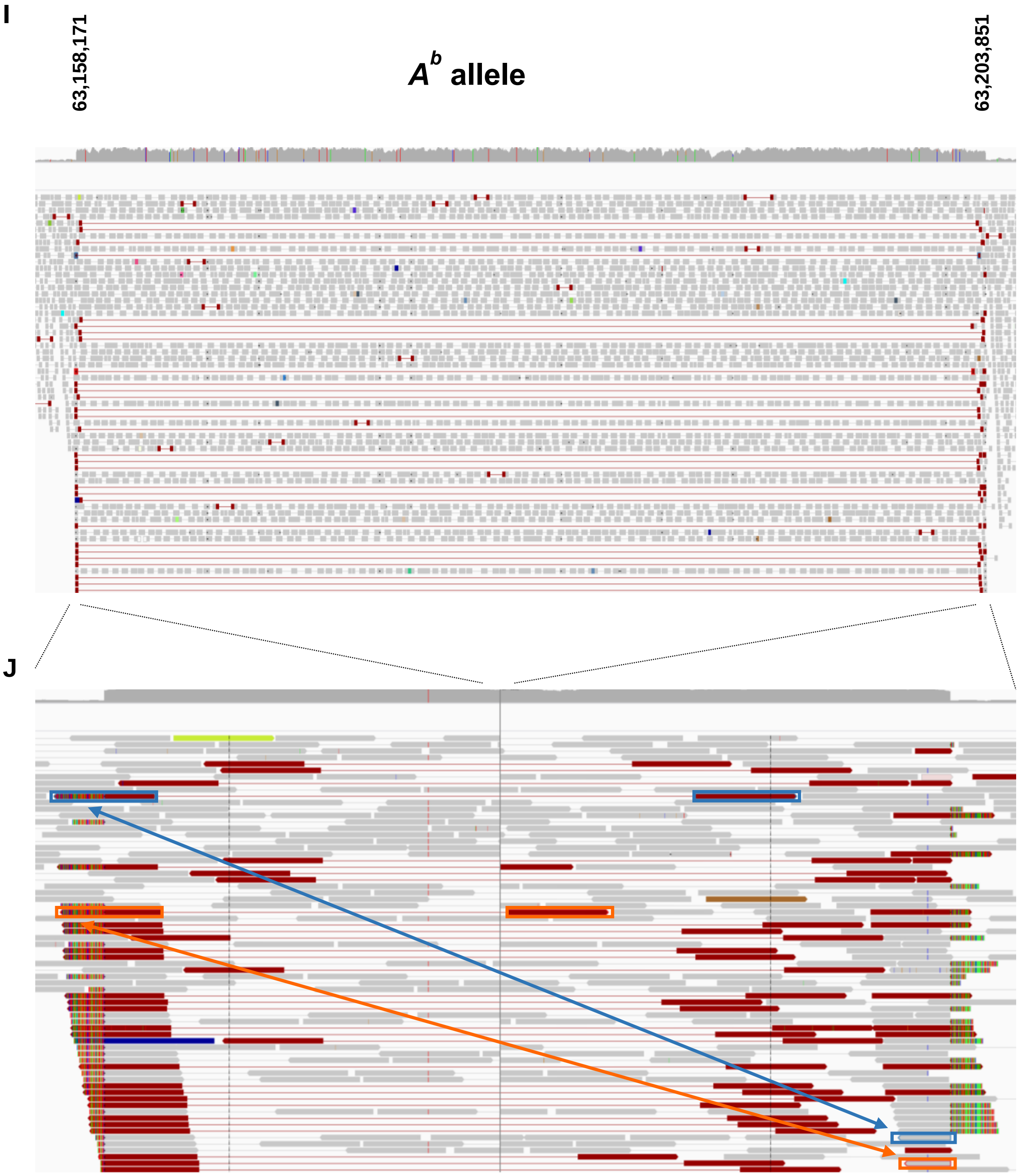

S4 Figure. Details of the CNV alleles. **I** IGV screenshot of the A^b allele derived from the whole genome sequence data on an individual St. Gallen Booted goat. The A^b allele contains 5 tandem copies of a ~45 kb sequence region. The illumina sequence reads are displayed as read-pairs. Several read-pairs that span from one into the next copy of the CNV are highlighted in red color. **J** Enlarged details of the breakpoints. Two read-pairs bridging individual copies of the amplified region are highlighted in blue and orange, respectively. The soft-clipped based at the left boundary align to the reference sequence at the right boundary of the amplified region. This confirms that the amplified copies are arranged in tandem in a head-to-tail orientation.

K

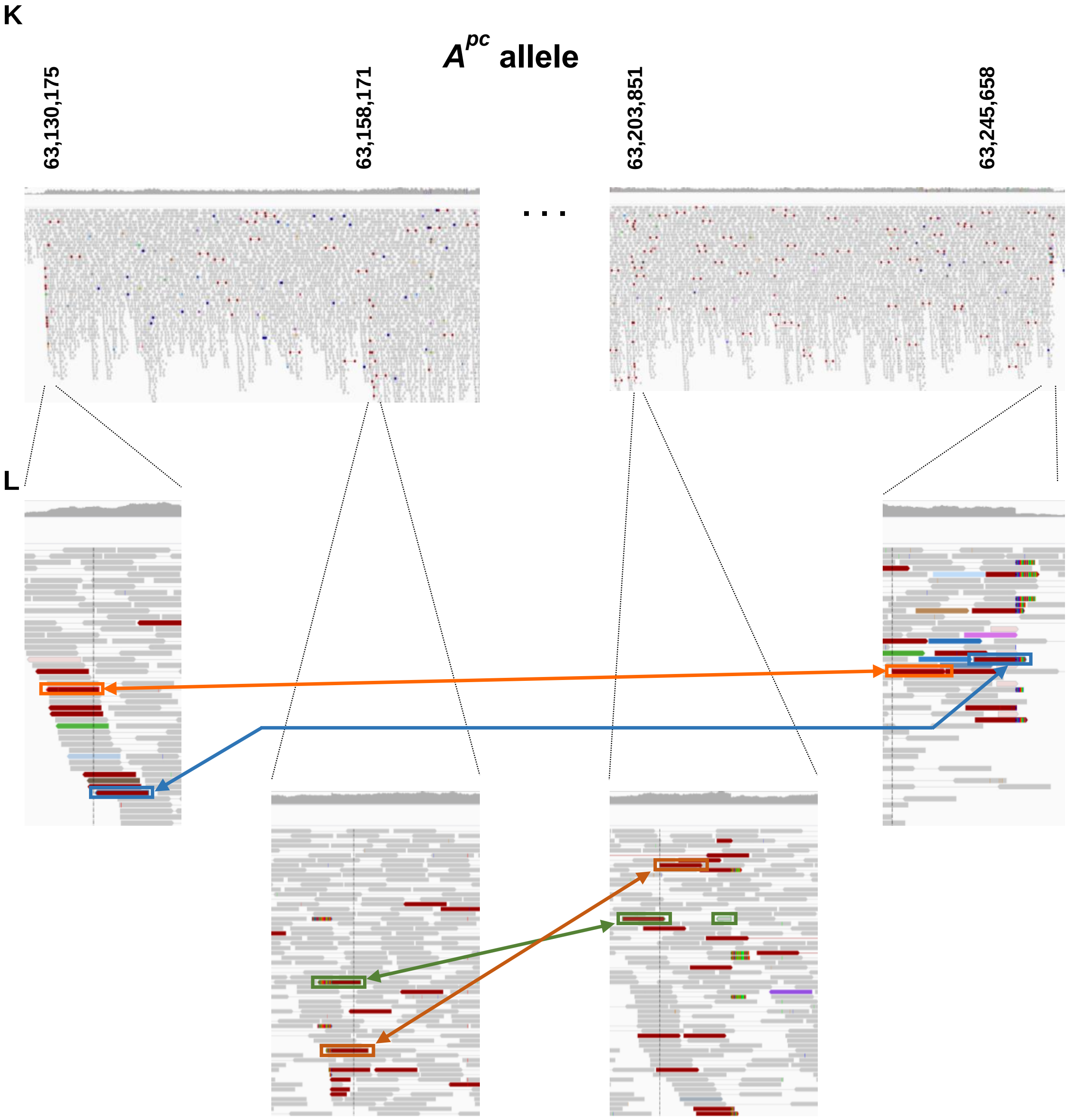

S4 Figure. Details of the CNV alleles. **K** IGV screenshot of the A^{pc} allele derived from the whole genome sequence data on an individual Peacock goat. The A^{pc} allele contains a central part with 4 tandem copies of a ~45 kb sequence region. The breakpoints of this central region are identical to the breakpoints of the A^{b} CNV allele. This central region is flanked by 3 tandem copies of a ~115 kb sequence region. **L** Enlarged details of the two pairs of breakpoints. Selected read-pairs bridging individual copies of the amplified region are highlighted in orange, blue, green and brown, respectively. This confirms that the amplified copies are arranged in tandem in a head-to-tail orientation.
