# Supplementary material for: Selection signatures in goats reveal copy number variants underlying breed-defining coat color phenotypes
Source: PLoS Genet. 2019 Dec 16;15(12):e1008536. doi: 10.1371/journal.pgen.1008536 (PMC6936872; doi:10.1371/journal.pgen.1008536)
Supplement: Attachment — Submitted filename: Reviewer_response_R1.pdf [file pgen.1008536.s012.pdf]

Response to Reviewers’ Comments

Reviewer #1:

The authors of this study have performed pooled whole genome sequencing of 20 goat breeds and identified thousands of putative signals of selection based on reduced heterozygosity. Here they report that some of the detected putative selective sweeps involve copy number variants at KIT and ASIP associated with coat color variants. The frequent occurrence of such CNVs is interesting. Further characterization of these CNVs would make the paper even more interesting for the readers of this journal.

Specific comments

1. It would be interesting to know to which extent the copy number varies between individuals within breeds. Since the authors report pooled sequencing data it is possible that the copy numbers here are just the average and that variation in copy number may contribute to heterogeneity in phenotypes. The authors have apparently some individual sequence data that can be directly used for this and it would be easy to design TaqMan assays to explore this.

Response: We expanded the S4 Table (which now became the S5 Table in the revised version) and give the exact copy numbers for all copy number-variable intervals in the 120 individually sequenced goats. The data do not conclusively indicate any variation in the absolute copy number of the individual CNV alleles between animals. There is minor variation in the relative coverage (e.g. from 7.4 to 9.9 in Grisons Striped goats homozygous for the A^{sm} allele). However, we think that these minor variations are more likely due to uneven distribution of the sequence reads rather than true differences in copy number. According to our experience, it is extremely challenging to determine exact absolute copy numbers in CNV alleles with relatively high copy numbers (e.g. to differentiate between alleles with 8 or 9 copies). Answering this question will require an additional major study that is beyond the scope of this manuscript.
The individual sequences revealed a few goats that are heterozygous for two different *ASIP* alleles (see the new S5 Table). This is an unexpected result as Swiss goat breeds are intensely selected for correct coat color. However, goat breeding is not monitored as closely as e.g. dairy cattle breeding and our data clearly show that not all Swiss goat breeds have reached complete fixation of their *ASIP* genotypes.

2. It would also be interesting to confirm that these are tandem duplications located at the KIT and ASIP loci and not translocated copies in other places of the genome. This can be confirmed by characterizing the duplication borders either using available short reads or by PCR. This would also allow the identification of the exact duplication breakpoints which can be used to characterize how the initial structural change

occurred. Furthermore, this would allow the authors to confirm that those cases where the authors assume that different alleles share a common origin and one allele has evolved further by accumulating additional changes on the same haplotype as suggested for the two KIT alleles (Fig. 2) and for A(pc).

Response: The available short read alignments indeed confirm the postulated tandem amplification in a head to tail orientation. We prepared an additional supplementary figure (S4 Figure) showing IGV screenshots with details of reads spanning the amplification breakpoints.

The available short read alignments also demonstrated that the KIT^{ANG} and KIT^{BAR} as well as the A^{b} and A^{pc} CNV alleles share amplification breakpoints. This suggested to us pairwise common origins of these *KIT* and *ASIP* alleles. We were not able to reconstruct the exact historical time course of the additional sequence changes leading to these alleles. The alleles differ at a few SNV positions within the amplified regions. We speculate that this may have been due to e.g. gene conversion events. We slightly revised the text to make this clear.

3. Another analysis worth doing is to explore the sequence haplotypes present in the duplicated copies, the two or more copies may be identical in sequence represent different alleles depending on how the duplication event occurred and how much sequence homogenization has taken place between the two copies. For instance, the authors report that there was not any increased homozygosity in white Saanen goats. The explanation could be that the 150 kb triplication shows sequence heterogeneity between copies which are interpreted as heterozygous position in the SNP analysis.
   The authors write on page 10 that A(pc) is probably derived from the A(b) allele, this can be tested by comparing the sequence identities across the CNV region and by determining the exact duplication breakpoints.

Response: We thank the reviewer for this valuable comment. Indeed, the individual copies in the A^{Wt} allele showed sequence differences, which decreased the signal in the -ZH_{p} analysis. We added this to the discussion.

We also modified our statements about the relation of the A^{b} and A^{pc} alleles (see comment above).

4. As far as I understand the coat color of the Peacock goat has not been reported before. It appears very likely that the CNV reported here is contributing to the phenotype but I assume it is impossible to tell if the phenotype described as A(pc) is solely determined by an ASIP allele. It may be a combined effect of this variant in combination with one or more other loci. I recommend that the authors state that this is a preliminary description of the Apc phenotype.

Response: We added a sentence to the discussion that explicitly mentions the possibility of additional modifiers.

5. Adalsteinsson et al described 8 ASIP alleles in goats, 5 alleles are characterized here, one of these is new, so the question is whether there are 4 more alleles not yet characterized or were these represented among the 20 different breeds but were not associated with a sweep signal and/or CNV?

Response: This is a very good question. Unfortunately, we cannot be sure whether any of the other *ASIP* alleles reported by Adalsteinsson is present in the goats of our study. We did not observe any additional selection signatures at the *ASIP* locus in the Swiss goat breeds. We speculate that only the non-agouti allele A^{a} (Adalsteinsson: "no pattern") is likely to be caused by a coding loss-of-function variant. All other mutant *ASIP* alleles are likely to be caused by non-coding regulatory variants. We were admittedly very lucky that several of these are apparently caused by large CNVs that are easy to identify. However, it is possible and even likely that some of the missing *ASIP* alleles are caused by more subtle sequence variation. It will be very challenging to identify e.g. a regulatory SNV that might be located far away from the coding sequence.

6. I disagree with the statement at the bottom of page 14 that white spotting in Pak Angora and Barbari goats are likely due to insufficient expression of KIT. The lesson from the KIT literature in mouse and other species is that KIT loss-of-functions in the heterozygous state gives a mild white spotting and homozygotes are completely white and lethal or sublethal due to pleiotropic effects on hematopoiesis. In contrast, structural changes often cause dominant white spotting (e.g. patch and rump-white in mouse) most likely due to ectopic expression of KIT interfering with KIT normal function disrupting melanocyte migration/maturation. I think it is much more likely that the CNVs reported here alters the KIT expression pattern than leading to just insufficient expression.

Response: We completely agree with the reviewer and revised the discussion accordingly.

Minor comments

1. Page 4, middle. To the best of my knowledge the ligand for MC1R is named melanocyte stimulating hormone (MSH). The 1 in melanocortin refers to that it is 1 out of 5 melanocortin receptors.

Response: Revised accordingly.

2. Page 9. There is redundancy between the figure legend and the text.

Response: We slightly modified the text and the figure legend. We apologize that there still is some redundancy. However, we feel that it is necessary to state some details of the complex rearrangement in the KIT^{BAR} allele. If these details are missing, either the main text or the figure on its own become incomprehensible.

Reviewer #2:

The authors used pooled sequencing and windowed selection scans to identify putative selective sweeps between 20 goat populations.

This is a very good, comprehensive analysis using an efficient method for selective sweep detection. However, there are some aspects of the approach and presentation that should be addressed.

1. The authors refer to a drop in pooled heterozygosity as a selective signature. This in itself is not, nor is strong FST. When combining the two, preferably coupled to a test for neutrality, one may consider a selective sweep. The authors should apply more formal tests for selection.

Response: We appreciate the comment by the reviewer. Unfortunately, we were not able to perform additional large-scale formal tests due to lacking human and computational resources. Our calculations of the pooled heterozygosity took several months on the servers of our University. We ask the reviewer to consider that the isolated analysis for reduced heterozygosity in pool-seq data has been applied in several publications in the past (e.g. Rubin *et al.* 2010 *Nature*; Rubin *et al.* 2012 *PNAS*). We added a sentence to the discussion emphasizing the limitations of our study.

2. 2,239 selection signatures, with over 100 per pool is quite high. How do the authors account for demographic factors driving divergence as opposed to positive selection, particularly since you have access to individual data from five breeds? Did they attempt other formal methods such as XP-EHH, XP-CLR, or iHS?

Response: As mentioned in the comment above, we cannot definitively distinguish between drift and positive selection. The suggested analyses using LD-based methods on the individual sequences will be highly interesting for future studies, but are beyond the scope of this manuscript. It would be very difficult to explain why we use different sets of methods for different breeds investigated in this manuscript. We have pool-seq data on 21 breeds, but individual sequence data on only 5 breeds. We are currently in the process of obtaining further individual sequences from other goat breeds (AdaptMap consortium) and plan to perform such analyses as soon as we have a complete and representative set of data.

3. It would be informative to include zFST with the zHp data so readers can assess if the authors' claims hold true. I see them referenced in Figure S1 and S3, but these were not included in the submission received by this reviewer – only supplemental tables.

Response: We sincerely apologize for not having uploaded the supplementary figures. They are now included with the revised version of the manuscript and should contain the requested information. We additionally prepared a new S4 Table listing the selection signatures identified by the F_{ST} analysis.

4. How much total genomic length do the 2,239 signatures represent?

Response: We now give the total length of the ZH_p and ZF_{ST} based selection signatures (1.1% and 0.4% of the genome, respectively).

5. Copy number changes distort selective signals since they are collapsed in the assembly, and typically contain interlocus variation. Thus, the presence of a CNV does not mean positive selection, particularly when variants are excluded based on coverage, which would distort Hp. The authors should compare these signals to true selective sweep signals driving non-copy related evolution. Do the copy number estimates hold true for individual sequences?

Response: We fully agree with the reviewer that CNVs distort the signals for selection. We revised and expanded the discussion with respect to this comment.
We must emphasize that the identification of 6 CNVs as most likely causative variants for observed selection signatures **came as a complete surprise to us**! We used a coding *TYRP1* single nucleotide variant to validate our experimental approach and this positive control worked perfectly fine.
For the question regarding the copy numbers in individual sequences, please see our response to comment 1 from reviewer 1.

6. Detecting sweeps is typically done using varying window sizes to test the robustness of the detected signal. Did the authors examine this, and choose 150kb based on the result? How did they determine this window size was appropriate for their data?

Response: We evaluated different window sizes (25 to 300 kb) based on Rubin *et al.* 2012. After reviewing the distributions, we concluded that similar to the study of Rubin *et al.* in pigs, 150 kb was the most appropriate window size, because this size yielded few windows with <20 SNPs, while retaining a theoretically appropriate length to detect smaller sweeps. We did not change the manuscript with respect to this comment as this was already described in the methods section of our initial submission.

7. Identifying CNV is a separate method from identifying selective sweeps. Was there an effort to identify CNV outside sweep regions? This should be included since the authors are trying tie CNV signals to those of selective sweeps.

Response: As mentioned above, the CNV identification was not the primary aim of our study. The aim of our study was to identify selection signatures and candidate causative genetic variants for specific coat color traits that are known to be under selection in several goat breeds. Our approach to identify candidate causative variants was completely open to any kind of sequence variation. It came as a surprise that we found so many CNVs.

8. In sum, the discovery of these specific CNV, and their effect on ASIP are convincing and will constitute a significant contribution to the field. However, the conflation of selection and copy number is confusing, and should be clarified further. Without a comprehensive description of CNV and comparison with Hp, FST, and a more formal test for selection, it is difficult to conclude this is selection, rather than simply demography.

Response: We agree with the reviewer that we have not provided formal proof for selection with the appropriate statistical test from population genetics. However, we would like to argue that the identification of plausible candidate variants within selection signatures "rescues" an imperfect mapping and is thus worth publishing.
We slightly modified the abstract and the results section to emphasize that the identification of the selection signatures was "only" a means and first step towards identification of the causal variation responsible for specific coat color traits.
